# Connectome‐Based Predictive Modeling of Trait Mindfulness

**DOI:** 10.1002/hbm.70123

**Published:** 2025-01-08

**Authors:** Isaac N. Treves, Aaron Kucyi, Madelynn Park, Tammi R. A. Kral, Simon B. Goldberg, Richard J. Davidson, Melissa Rosenkranz, Susan Whitfield‐Gabrieli, John D. E. Gabrieli

**Affiliations:** ^1^ McGovern Institute for Brain Research Massachusetts Institute of Technology Cambridge Massachusetts USA; ^2^ Department of Brain and Cognitive Sciences Massachusetts Institute of Technology Cambridge Massachusetts USA; ^3^ Department of Psychological & Brain Sciences Drexel University Philadelphia Pennsylvania USA; ^4^ Center for Healthy Minds University of Wisconsin–Madison Madison Wisconsin USA; ^5^ Department of Counseling Psychology University of Wisconsin–Madison Madison Wisconsin USA; ^6^ Department of Psychology University of Wisconsin–Madison Madison Wisconsin USA; ^7^ Department of Psychiatry University of Wisconsin–Madison Madison Wisconsin USA; ^8^ Department of Psychology Northeastern University Boston Massachusetts USA; ^9^ Center for Precision Psychiatry, Department of Psychiatry Massachusetts General Hospital Boston Massachusetts USA

**Keywords:** attention, connectome, multi‐site, predictive models, resting‐state fMRI, trait mindfulness

## Abstract

Trait mindfulness refers to one's disposition or tendency to pay attention to their experiences in the present moment, in a non‐judgmental and accepting way. Trait mindfulness has been robustly associated with positive mental health outcomes, but its neural underpinnings are poorly understood. Prior resting‐state fMRI studies have associated trait mindfulness with within‐ and between‐network connectivity of the default‐mode (DMN), fronto‐parietal (FPN), and salience networks. However, it is unclear how generalizable the findings are, how they relate to different components of trait mindfulness, and how other networks and brain areas may be involved. To address these gaps, we conducted the largest resting‐state fMRI study of trait mindfulness to‐date, consisting of a pre‐registered connectome‐based predictive modeling analysis in 367 meditation‐naïve adults across three samples collected at different sites. In the model‐training dataset, we did not find connections that predicted overall trait mindfulness, but we identified neural models of two mindfulness subscales, *Acting with Awareness* and *Non‐judging*. Models included both positive networks (sets of pairwise connections that positively predicted mindfulness with increasing connectivity) and negative networks, which showed the inverse relationship. The *Acting with Awareness* and *Non‐judging* positive network models showed distinct network representations involving FPN and DMN, respectively. The negative network models, which overlapped significantly across subscales, involved connections across the whole brain with prominent involvement of somatomotor, visual and DMN networks. Only the negative networks generalized to predict subscale scores out‐of‐sample, and not across both test datasets. Predictions from both models were also negatively correlated with predictions from a well‐established mind‐wandering connectome model. We present preliminary neural evidence for a generalizable connectivity models of trait mindfulness based on specific affective and cognitive facets. However, the incomplete generalization of the models across all sites and scanners, limited stability of the models, as well as the substantial overlap between the models, underscores the difficulty of finding robust brain markers of mindfulness facets.

## Graphical abstract

Trait mindfulness subscales—*Acting with Awareness* and *Non‐judging—*were predicted by connectome models trained on resting‐state fMRI data.

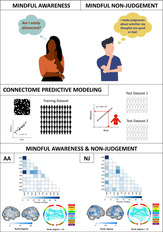

**Summary**

- This large multi‐site study finds the first evidence of generalizable brain differences associated with higher or lower mindfulness across individuals.
- Somatomotor, visual and DMN network connectivity featured prominently in predicting mindfulness.
- Brain differences were not found across all sites, and reliability over fMRI sessions was low, underscoring the difficulty of finding a “mindful brain.”

## 1 Introduction

Mindfulness, often defined as the act of paying attention to the present moment without judgement (Bishop et al. 2004), is a construct that has been researched intensely in recent years. Western psychological theories of mindfulness propose that mindfulness varies in a meaningful, reliable way across individuals (Baer et al. 2006; Brown and Ryan 2003; Rau and Williams 2016), and that this “trait mindfulness” is a malleable quality that can be trained through repeated states of mindfulness (e.g., meditation) (Kiken et al. 2015; Quaglia et al. 2016). Trait mindfulness is a multidimensional construct that encompasses paying attention to the present moment, relating to thoughts and feelings in an accepting way, and more. Trait mindfulness is often measured by self‐report scales, such as the Five Facet Mindfulness Questionnaire (FFMQ) (Baer, Smith, and Allen 2004) and the Mindful Attention Awareness Scale (MAAS) (Brown and Ryan 2003). Greater trait mindfulness has been frequently associated with a range of positive mental health outcomes, including more positive affect, improved self‐compassion, greater openness to experience, and better quality of life (Allen, Romate, and Rajkumar 2021; Amundsen et al. 2020; Chu and Mak 2020; Kong, Wang, and Zhao 2014; Schutte and Malouff 2011), and negatively associated with outcomes like negative affect, stress, and anxiety (Carpenter et al. 2019; Coffey and Hartman 2008; de Bruin, Zijlstra, and Bögels 2014; Greco, Baer, and Smith 2011; Tomlinson et al. 2018; Treves et al. 2023). Given the importance of trait mindfulness as a predictor of mental health, there is a clear need to understand its neural underpinnings. Neuroimaging of trait mindfulness could help us understand mental health conditions (Zhuang et al. 2017), reveal pathways of action in mindfulness interventions (Goldberg et al. 2019), and provide possible targets for neuromodulation (Cain et al. 2024; Zhang et al. 2023). Motivated by these possibilities, this study investigated the functional neuroimaging basis of trait mindfulness.

Resting‐state functional magnetic resonance imaging (fMRI) data, measured when participants lie awake in the fMRI scanner in a task‐free state, may provide brain‐based measures correlating with trait mindfulness. A resting state does not explicitly engage cognitive or emotional processes. Instead, it is typically used to study correlated intrinsic brain signals, or functional connectivity, while a participant is at rest. These correlations, which can be reliable given sufficient data (Finn et al. 2015; Laumann et al. 2015; Noble et al. 2017), are thought to reflect stable aspects of individual functional brain organization (Shen et al. 2017; Smith et al. 2013). In addition, resting state data are relatively easy to collect and can be compiled in large online databases to increase sample size (Biswal et al. 2010; Eickhoff et al. 2016; Poldrack and Gorgolewski 2017).

Static functional connectivity (SFC) is measured by correlations between brain regions over the course of a resting‐state fMRI scan, and several networks of correlated brain regions are plausibly related to variation in trait mindfulness. One network is the default‐mode network (DMN), involved in internally focused, self‐referential processing, and consisting of brain areas such as the precuneus, posterior cingulate, and ventromedial prefrontal cortex (Raichle et al. 2001). Two other candidate networks are the salience network (SN), involved in stimulus‐driven attention and including the insula and mid‐cingulate (Seeley et al. 2007); and the frontoparietal network (FPN), involved in externally focused, goal‐directed attention and consisting of lateral frontal and parietal areas (Dosenbach et al. 2008; Greicius et al. 2003; MacDonald et al. 2000).

Despite at least nine studies on resting‐state SFC and trait mindfulness (Bilevicius, Smith, and Kornelsen 2018; Doll et al. 2015; Harrison et al. 2019; Hunt et al. 2022; Kong et al. 2016; Li et al. 2022; Parkinson, Kornelsen, and Smith 2019; Shaurya Prakash et al. 2013; Wang et al. 2014), there is no consistent relation between these networks and trait mindfulness. For example, Bilevicius, Smith, and Kornelsen (2018) found that *decreased* connectivity of the SN and the cuneus (often considered part of the DMN) correlated with MAAS total scores, but Parkinson, Kornelsen, and Smith (2019), found that *increased* connectivity of the SN and cuneus correlated with FFMQ total scores. Both studies were conducted with *n* ~ 30 participants. Some studies found that trait mindfulness correlated with reduced within‐DMN connectivity (Bilevicius, Smith, and Kornelsen 2018; Doll et al. 2015; Harrison et al. 2019; Wang et al. 2014), but a subsequent larger sample study (*n ~* 100) failed to replicate this finding (Hunt et al. 2022). Sources of variability between studies could stem from variable sample characteristics, small sample sizes, different methodologies (i.e., choice of seed regions), or a lack of test–retest reliability in the fMRI measures. A broader concern is that mindfulness may not be a unitary trait (Altgassen, Geiger, and Wilhelm 2023; Beloborodova and Brown 2023) and, therefore, it is unlikely to involve unitary brain processes. It may be that mindfulness involves related but distinct subcomponents like attention and non‐judgement (Bishop et al. 2004). Indeed, the FFMQ was developed based on factor analyses of previous mindfulness questionnaires, resulting in statistically dissociable facets of *Acting with Awareness* (*AA*), *Non‐judging* (*NJ*), *Non‐reactivity* (*NR*), *Describing* (*D*), and *Observing* (*O*) (Baer et al. 2006, 2008; although *Observing* may show limited validity, Gu et al. 2016). *AA* refers to attending to one's experiences and actions, for example, “It seems I am running on automatic without much awareness of what I'm doing” (reverse‐coded). *NJ* relates to not judging one's thoughts or emotions, for example, “I criticize myself for having irrational or inappropriate emotions” (reverse coded). *Non‐reactivity* involves observing thoughts without being caught up in them, for example, “I perceive my feelings and emotions without having to react to them.” *Describing* refers to labeling emotions with words, for example, “I am good at finding words to describe my feelings.” Last, *Observing* is defined as noticing bodily sensations, for example, “I pay attention to sensations, such as the wind in my hair or sun on my face.” These individual components may relate to different patterns of resting‐state brain connectivity.

Here we addressed these concerns in a preregistered, multisite study of resting‐state fMRI and trait mindfulness to date. The present sample (*n* = 367 meditation‐naïve adults) constitutes the largest sample size of any laboratory‐based neuroimaging study of trait mindfulness (for a systematic review, see (Treves, Pichappan, et al. 2024). We used a data‐driven, whole‐brain approach called connectome‐based predictive modeling (CPM). CPM tests pairwise connections across the whole brain (Shen et al. 2017), and can find positive and negative network models that predict individual differences. A key feature of CPM is prediction—whereas correlation may inflate the strength of an association, prediction of held‐out data is more accurate (Gabrieli, Ghosh, and Whitfield‐Gabrieli 2015). CPM has proven predictive power for individual differences in IQ, creativity, sustained attention, mind‐wandering (MW), and other traits (Beaty et al. 2018; Finn et al. 2015; Kucyi et al. 2021; Rosenberg et al. 2015). In this study, we used CPM to investigate relationships between trait mindfulness, assessed with the FFMQ, and resting‐state SFC in functional networks across the whole brain (including the DMN, FPN, and SN). We assessed whether the relationships generalized to independent samples, and we examined the relations of brain networks to the overall FFMQ score as well as the five FFMQ facets. We hypothesized a priori that mindfulness would be predictable by brain measures, but did not propose specific brain areas, or which facets would be predictable, given the inconsistencies in the previous literature.

## 2 Materials and Methods

We preregistered this study before analysis at https://osf.io/dtk9a/. All deviations are reported in the Data S1.

### 2.1 Training Dataset: Wisconsin

We obtained imaging and phenotypic data from the University of Wisconsin–Madison meditation study (NCT02157766). The sample consisted of 206 meditation‐naïve participants (age *M* = 30.9, *SD* = 13.1 years, 85 male) who completed an eyes‐open resting‐state scan and the FFMQ (Baer et al. 2006). Of those 206 participants, 71 had asthma, and the full sample was retained. One of the aims of the original trial was to evaluate the relationship between psychological factors and asthma, but this aim was not relevant to the present study. Asthma status was controlled for in our analyses by conducting partial correlations with an indicator variable, given evidence for relationships between chronic inflammatory conditions and functional connectivity (Aruldass et al. 2021; Labrenz et al. 2019) as well as mental health (Stanescu et al. 2019). No participants had psychiatric diagnoses.

Images were acquired on a GE MR750 3.0 Tesla MRI scanner with a 32‐channel head coil. Anatomical scans consisted of a high‐resolution 3D T1‐weighted inversion recovery fast gradient echo image (450 ms inversion time; 256 × 256 in‐plane resolution; 256 mm field of view [FOV]; 192 × 1.0 mm axial slices). A 12 min functional resting‐state scan run was acquired using a gradient echo echo‐planar imaging (EPI) sequence (360 volumes; repetition time [TR]/echo time [TE]/Flip, 2000/20 ms/75°; 224 mm FOV; 64 × 64 matrix; 3.5 × 3.5 mm in‐plane resolution; 44 interleaved sagittal slices; 3 mm slice thickness with 0.5 mm gap). The in‐plane resolution was decreased after the first 21 participants from 3.5 × 3.5 to 2.33 × 3.5 mm to better address sinus‐related artifacts, resulting in a matrix of 96 × 64. Resolution change was controlled for in subsequent analyses by partialling out an indicator variable.

### 2.2 Test Dataset: Stanford Science of Behavior Change

We obtained imaging and phenotypic data from the Stanford Science of Behavior Change project (https://scienceofbehaviorchange.org/projects/poldrack‐marsch/) (Bissett et al. 2024). The sample consisted of 82 meditation‐naïve participants (age *M* = 23.6, *SD* = 4.9 years, 27 male) who completed an 8‐min eyes‐open resting state scan and the FFMQ (Baer et al. 2006). Of those 82 participants, 22 had diagnoses of anxiety, depression, or other clinical conditions, and all participants were retained. To control for the influences of clinical conditions, modelling approaches employed partial correlations. When removing the clinical participants, results mirrored the partial correlation findings.

Participants were scanned in a GE Discovery MR750 3‐Tesla system with a 32‐channel Nova Medical head coil at the Stanford center for Cognitive and Neurobiological Imaging. The T1‐weighted scan used a BRAVO sequence with the following parameters: duration (4 min and 50 s), TR (7.24 ms), TE (2.784 ms), flip angle (12°), slice number (186), and resolution (0.9 mm isotropic voxels). The T2*‐weighted, gradient‐echo echo‐planar imaging, scan parameters were as follows: duration (8 min), multiband acceleration factor (8), TR (0.68 s), TE (30 ms), flip angle (53°), echo spacing (0.57 ms), slice number (64), resolution (2.2 mm isotropic), and phase encoding anterior to posterior.

### 2.3 Test Dataset: Leipzig Mind–Brain–Body

We downloaded openly available imaging and phenotypic data from the functional connectome phenotyping dataset (Babayan et al. 2019), a component of the MPI‐Leipzig Mind–Brain–Body project (Mendes et al. 2019). Procedures for this study were approved by the ethics committee at the medical faculty of the University of Leipzig (097/15‐ff). The sample consisted of 79 meditation‐naïve participants (modal age range 20–25, 45 male) who completed four eyes‐open resting‐state scans and completed the FFMQ (Baer et al. 2006), translated to German. No participants had psychiatric diagnoses.

Participants were scanned in a 3‐Tesla Siemens Magnetom Verio system with a 32‐channel head coil at the University of Leipzig. The T1‐weighted, 3DMP2RAGE, scan parameters were as follows: duration (8.22 min), TR (5 s), TE (2.92 ms), flip angle 1/2 (4/5°), TI 1/2 (700/2500 ms), slice number (176), resolution (1.0 mm isotropic). The T2*‐weighted, gradient‐echo echo‐planar imaging, scan parameters were as follows for each of the four runs: duration (15 min 30 s), multiband acceleration factor (4), TR (1.4 s), TE (39.4 ms), flip angle (69°), echo spacing (0.67 ms), slice number (64), and resolution (2.3 mm isotropic). In the first and third runs, the phase encoding direction was anterior to posterior, whereas in the second and fourth runs, the phase encoding direction was posterior to anterior.

### 2.4 Measures

The FFMQ consists of 39 questions, corresponding to five statistically separable subscales: *AA, NJ, NR, D*, and *Observing (O)* (Baer et al. 2006, 2008). Each question on the FFMQ is rated on a 5‐point Likert scale, ranging from “1 = Never or very rarely true” to “5 = Very often or always true”. The *AA, NJ*, and *D* subscales include reverse‐scored questions. The lowest possible total score is 39 and the highest possible score is 195, with higher scores representing higher levels of mindfulness. We used the total scores, the total scale without observing (Baer, Gu, and Strauss 2022; Gu et al. 2016; Pang and Ruch 2019), and the subscales. The FFMQ has demonstrated acceptable internal consistency across a range of samples (0.72–0.92, Baer et al. 2008). We assessed relationships between the subscales using Pearson's correlations in the Wisconsin dataset. For comparisons of the total FFMQ scores between the datasets, we conducted simple unpaired, heteroskedastic *t*‐tests given the large sample sizes. We reported effect sizes using Cohen's *d*.

### 2.5 Procedure

Preprocessing was identical to Kucyi et al. (2021) and details are provided. We preprocessed each fMRI run individually using the same procedures across datasets, based on procedures implemented in the CONN toolbox (version 21a [https://www.nitrc.org/projects/conn]) (Whitfield‐Gabrieli and Nieto‐Castanon 2012) and SPM12 in Matlab R2019a (Mathworks Inc., Natick, MA). Preprocessing steps included deletion of the first four volumes, realignment and unwarping (Andersson et al. 2001), and identification of outlier frames (frame‐wise displacement > 0.9 mm or global BOLD signal change > 5 SD) (Nieto‐Castanon 2020). Functional and anatomical data were normalized into standard MNI space and, in a unified step, segmented into gray matter, white matter (WM), and cerebrospinal fluid (CSF) (Ashburner and Friston 2005). Smoothing of fMRI data consisted of spatial convolution with a Gaussian kernel of 6 mm full‐width half‐maximum (FWHM).

fMRI denoising involved linear regression of the following parameters from each voxel: (a) five noise components each from minimally eroded WM and CSF, respectively, based on aCompCor procedures (Behzadi et al. 2007; Chai et al. 2012) (b) 12 motion parameters (three translation, three rotation, and associated first‐order derivatives); (c) all outlier frames identified within participants; and (d) linear BOLD signal trend within session. After nuisance regression (Hallquist, Hwang, and Luna 2013), data were bandpass filtered to 0.008–0.09 Hz. Denoising procedures have been shown to reduce the impact of head motion on functional connectivity (Muschelli et al. 2014), but excessive head motion may confound estimates (Power, Schlaggar, and Petersen 2015; Siegel et al. 2017). To avoid this possibility, we excluded participants with mean overall frame‐wise displacement (FD) of > 0.15 mm (based on the Jenkinson method; Jenkinson et al. 2002) for the Wisconsin dataset and Stanford dataset. In the Leipzig dataset in which four rs‐fMRI runs were obtained within participants, we removed runs with more than 0.15 mm of mean FD, and participants based on the mean across runs. The final dataset sizes may be found in Table 1.

**TABLE 1:** The number of subjects in each dataset before and after head motion removal. We excluded participants with mean overall FD of > 0.15 mm (based on the Jenkinson method; Jenkinson et al. 2002) for the Wisconsin dataset and Stanford dataset. In the Leipzig dataset in which four rs‐fMRI runs were obtained within participants, we removed runs with more than 0.15 mm of mean FD, and participants based on the mean across runs. Please note that controlling for continuous values of head motion was also conducted using partial correlations.

Dataset	Total subjects	After head motion removal
Training: Wisconsin	206	188
Test: Leipzig	79	75
Test: Stanford	82	82

In addition, given that FD can influence observed relationships between functional connectivity and behavior (Siegel et al. 2017), we controlled for FD in analyses focused on relationships between functional connectivity and FFMQ scores (see Section 2.7).

### 2.6 Functional Connectivity Feature Extraction

For each individual, we extracted the preprocessed BOLD time series from the mean across all voxels within each node defined based on an intrinsic functional network atlas in MNI space, specifically, the Shen atlas of 268 whole‐brain regions (Shen et al. 2013). This atlas has been frequently used in CPM studies (e.g., Kucyi et al. 2021; Rosenberg et al. 2015). We computed Fisher z‐transformed Pearson correlation coefficient of time series, giving a matrix of functional connectivity values between all region pairs. We define region pairs as connections or edges.

### 2.7 Predictive Modeling Analysis

We chose the Wisconsin sample for training as it is the largest of the datasets in this study (Poldrack, Huckins, and Varoquaux 2020). We performed CPM using publicly available code (https://github.com/DynamicBrainMind/CPM_CONN). For each participant in the Wisconsin sample, we generated model‐based predictions of FFMQ or FFMQ subscales based on data from all other included participants, that is, leave‐one‐participant‐out cross‐validation (LOOCV). In each cross‐validation fold, we computed the Pearson correlation between each unique edge in the functional connectivity matrix (derived from the Shen atlas) and participant FFMQ scores. The resulting *r* values were statistically thresholded at *p* < 0.01 and separated into what is defined as a positive network (edges whose strength indexed higher FFMQ across subjects) and a negative network (edges whose strength indexed lower FFMQ scores). Both networks are binary masks. For each participant, we summed all *r* values in the positive network and the negative network, separately. We also calculated a single network strength value, the subtraction of negative network sums and positive network sums (Hu, Zhang, and Feng 2023; Kucyi et al. 2021; Rosenberg et al. 2020). Finally, we fit a linear model, based on all participants within the fold, of the form:

$$\text{FFMQ}=\beta^{*}\text{network}\_\text{strength}+c$$

In order to assess whether predicted versus observed scores in LOOCV held‐out participants were statistically significant at the group level, we generated a distribution of null values. To do so, we repeated all of the described CPM procedures, except the participant assignments of the FFMQ scores were randomly permuted (1000 iterations) to generate null correlation values. To compute a *p‐*value, we then calculated the probability of finding a null correlation at or above the true correlation (predicted versus observed FFMQ).

We repeated CPM procedures controlling for head motion by calculating partial correlations (*partialcorr* in MATLAB) between predicted and observed FFMQ. Using these partial correlations, we controlled for head motion, defined as the mean FD value per participant. We did not conduct permutations for the partial correlation tests because effect sizes were comparable to those obtained in the main analyses. We also controlled for participant asthma status by assessing partial correlations. Finally, we repeated CPM procedures with 10‐fold CV, which is a preferred approach for sample sizes > 100 (Poldrack, Huckins, and Varoquaux 2020).

### 2.8 Model Selection

We trained models for each of the seven FFMQ scores (Total, Total w/o Observe, and five subscales). Models were selected for generalizability testing based on an uncorrected *p* threshold < 0.05 based on the permutation testing (for similar approaches, see Kim et al. 2023a; Lee et al. 2021). In our predictive modelling framework, evidence for the model is evaluated by performance on the independent test dataset, not the statistical significance in the training dataset (Poldrack, Huckins, and Varoquaux 2020; Scheinost et al. 2019). Our main subsequent analyses focus only on the selected models. For external validation analyses assessing generalizability in the other dataset, as well as analyses of edge network identities, we computed CPM parameters and positive and negative masks based on data from *all* participants in the Wisconsin sample (i.e., a single fold).

### 2.9 Validation in Test Samples

We took the selected trained models and applied them on the Leipzig resting‐state data (averaged across runs, excluding runs with head motion, as described previously) and Stanford resting‐state data (excluding participants with head motion). Each functional connectivity matrix was masked with the positive and negative masks, and then those FC values from the selected edges were either applied independently or summed to form a network strength value. The connectivity predictor or network strength (dimensionality of one) was then used in the linear model to predict the FFMQ scores. We compared FFMQ predicted and observed values using Pearson's correlation, as well as mean squared error where appropriate. We also conducted partial correlations controlling for FD values.

### 2.10 Test–Retest Stability

There are some indications that CPM predictions may be more reliable and stable than individual edges (Taxali et al. 2021). To test this, we leveraged the Leipzig dataset, which had multiple 15‐min runs. We examined Pearson's correlations between the CPM network strengths for the first two runs and the last two runs. Additionally, we randomly selected 1000 individual edges, and estimated the probability of the CPM correlation compared to the distribution of random edges.

### 2.11 Analysis of Functional Connectivity Patterns Contributing to Mindfulness CPMs

To gain insight into the neuroanatomical patterns that contributed to the CPMs, we examined brain networks, nodes, and regions. The principal measure for display is “degree,” where a high degree means that a node/network/region is involved in many edges. First, we used the WASHU network labels to assign nodes to 10 networks for visualization (Power et al. 2011). The WASHU networks consist of SMN: somatomotor network, CO: cingular‐opercular network, AUD: auditory network, DMN: default‐mode network, VIS: visual network, FPN: frontoparietal network, SAL: salience network, SUB: subcortical network, VAN: ventral attention network, and DAN: dorsal attention network. A proportion of the nodes are not assigned to a network. Despite this limitation, the WASHU network labels were chosen because they include networks of interest (FPN, DMN, SAL, SMN, and VIS). We examined the number of connections within each network in matrix plots. Further, we plotted the specific node “degrees” on the brain medial and lateral surfaces using BioImage Suite (https://bioimagesuiteweb.github.io/webapp/connviewer.html). Finally, connectograms were plotted to display connections between brain regions using BioImage Suite (Data S1).

Comparisons of models were assessed, specifically the overlaps between edges selected by the models. We conducted non‐parametric permutation tests (shuffling the edges) to assess whether the degree of overlap was higher than chance.

### 2.12 Comparison to MW CPM

Previous work has identified a connectome‐based model that predicts mind‐wandering ratings within‐ and across‐individuals (MW‐CPM; Kucyi et al. 2021). As individuals who report higher MW may report less mindfulness (Mrazek, Smallwood, and Schooler 2012; Mrazek et al. 2013), we tested whether overall network strengths from the MW‐CPM model when computed on the current datasets correlated with overall network strengths from our mindfulness models.

### 2.13 Sensitivity Analyses

We conducted sensitivity analyses to identify whether different analysis approaches lead to improvements in generalizability. First, we considered whether generalization to datasets acquired with a different MRI scanner type may be a high bar. Thus we combined the data across scanners before shuffling and conducting an 80–20 training‐test split. In one analysis of this combined data, we conducted partial correlations using the means of the FFMQ within each dataset to control for dataset differences. Second, using the combined data, we examined whether other predictive modeling methods previously used in neuroimaging applications were more powerful than CPM, including tangent parameterization of connectivity, Brain Basis Sets and Elastic Net Regression. See Data S1 for Full Methods.

## 3 Results

### 3.1 Behavioral Measures

The mean FFMQ in the Wisconsin sample was 134.8 (*SD* = 17.8), in the Stanford sample 126.8 (*SD* = 17.4), and the in the Leipzig sample 107.2 (*SD* = 11.3) (Figure S1). Wisconsin FFMQ scores were significantly higher than Stanford scores as assessed by a two‐tailed Welch's *t*‐test (*t*(153.46) = 3.50, *p < 0*.001, Cohen's *d* = 0.44), and Leipzig's (*t*(221.96) = 15.45, *p < 0*.001, Cohen's *d =* 1.35). Stanford FFMQ scores were significantly higher than Leipzig FFMQ scores (*t*(139.68) = 8.49, *p < 0*.001, Cohen's *d =* 1.10). This difference in the trait measures could be related to the student sample of the Stanford dataset, or the German translation of the FFMQ, or cultural differences. FFMQ scores may vary across different samples (e.g., Goldberg et al. 2016; Isbel et al. 2020). Correlations between the subscales were significant but were all less than *r* = 0.5, indicating some independence (see Table S1). Subscales correlated with the total FFMQ score, *rs* > 0.6, and showed differences across sites similar to total FFMQ score differences (Figures S2 and S3).

### 3.2 Learning the Neural Features From the Training Dataset

**FIGURE 1:**
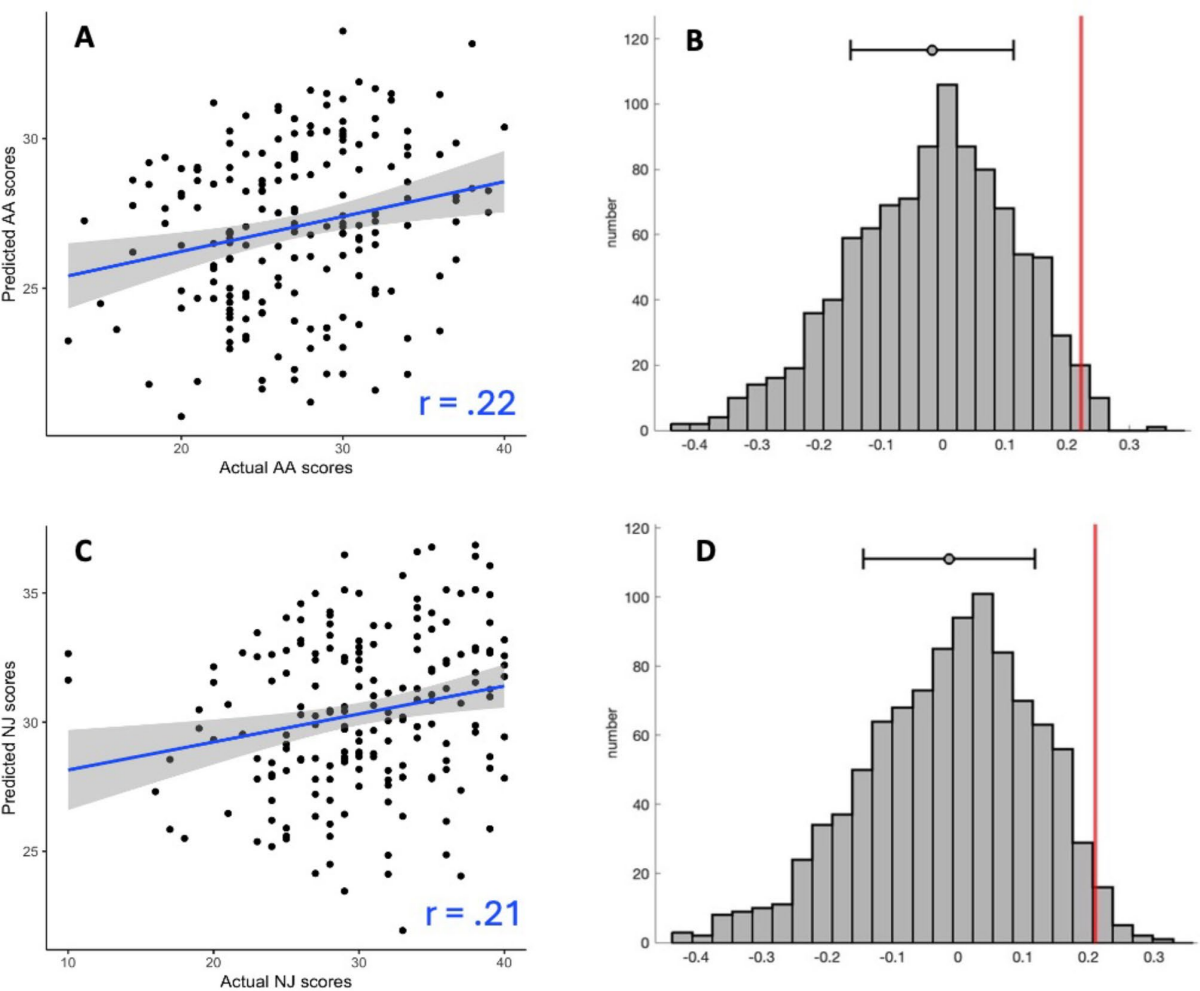
Prediction performance in the training set. (A) Predicted vs. observed values from leave‐one‐out cross validation, for *Acting with Awareness* subscale. (B) Correlation coefficient (in red) compared to the distribution of null correlation coefficients, for *Acting with Awareness* subscale. (C) Predicted vs. observed values from leave‐one‐out cross validation, for *Non‐judging* subscale. (D) Correlation coefficient (in red) compared to the distribution of null correlation coefficients, for *Non‐judging* subscale. In plots A and C, grey shading reflects 95% confidence intervals. In plots B and D, the mean and standard deviations are shown above the distributions.

We trained seven CPMs in the Wisconsin dataset, one for each subscale, the total score, and the total without observing. Eighteen participants were removed due to above‐threshold head motion. Full training set performance is reported in Table S2. The models predicting AA, and NJ showed positive correlations (Figure 1) between overall network strength (positive network—negative network) and the respective subscale (AA: *r*(186) = 0.22, NJ: *r*(186) = 0.21). The two models had non‐parametric (permutation testing) *p* values of 0.017 and 0.025, respectively, so we selected them for model testing in held out data. When using 10‐fold cross‐validation instead of leave‐one‐out cross‐validation (LOOCV), the results were similar (AA: *r*(186) = 0.16, *p* = 0.046; NJ: *r*(186) = 0.22, *p =* 0.021). The single‐fold AA model, which we call the AA‐CPM, consisted of 328 positive edges and 758 negative edges. Positive edges were present in 95.0% of LOOCV folds, and negative edges were present in 93.0% of LOOCV folds. Partial correlations with framewise‐displacement as a covariate showed similar effect sizes (*r*(186) = 0.22), indicating no influence of head motion on model prediction. Partial correlations with asthma status likewise resulted in similar effect sizes (*r*(186) = 0.21). The single‐fold NJ model, which we call the NJ‐CPM, consisted of 664 positive edges and 628 negative edges. Positive edges were present in 94.3% of LOOCV folds, and negative edges were present in 93.6% of LOOCV folds. Partial correlations with framewise‐displacement and asthma status as covariates were similar (*r*s of 0.21 and 0.19, respectively).

### 3.3 Features in the AA‐CPM

**FIGURE 2:**
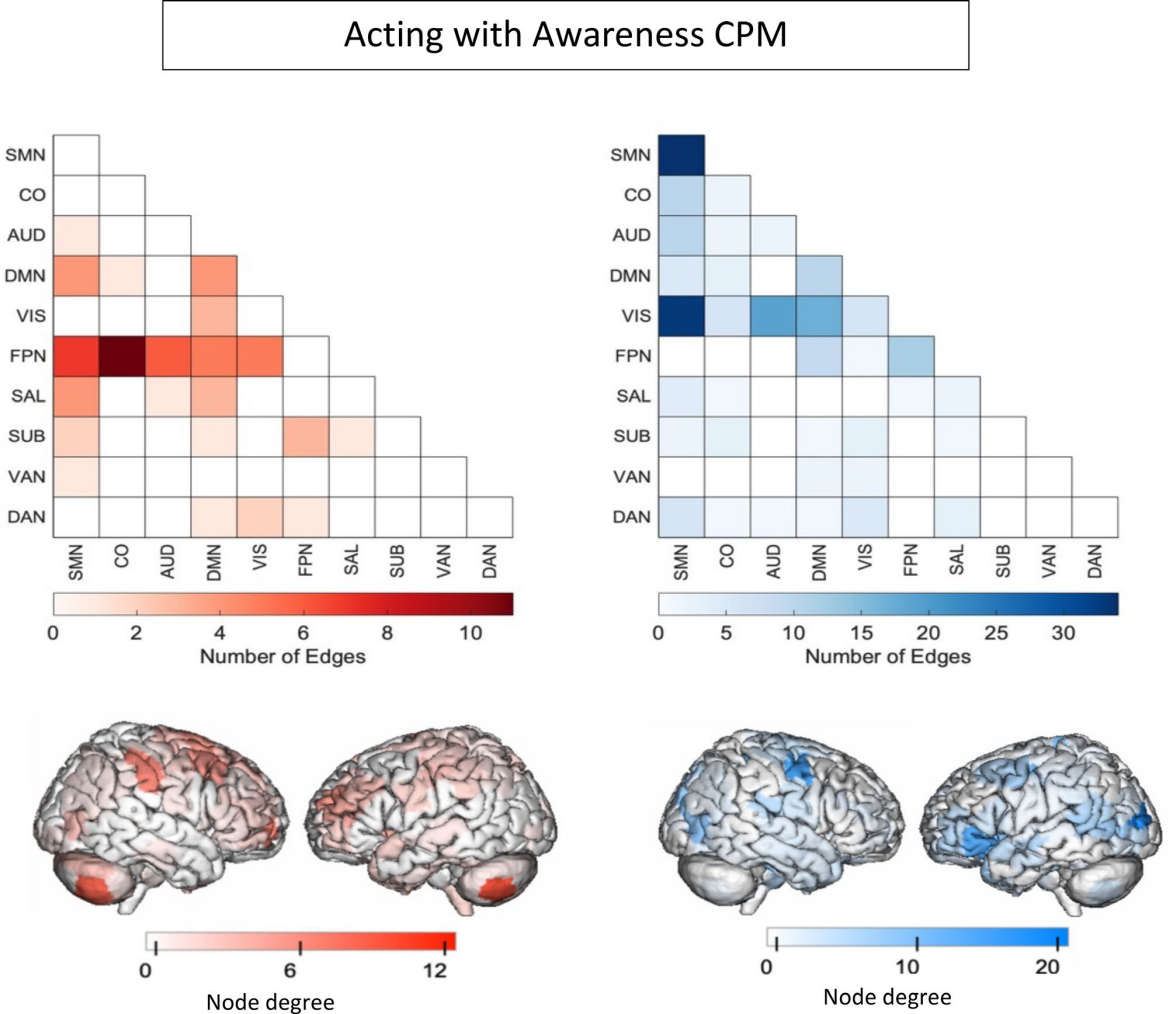
Edges included in single‐fold model of *Acting with Awareness* subscale of FFMQ using Shen atlas. In red are edges that positively predict *Acting with Awareness*. In blue are edges that negatively predict *Acting with Awareness*. AUD: auditory network, CO: cingular‐opercular network, DAN: dorsal attention network, DMN: default‐mode network, FPN: frontoparietal network, Node degree: number of connections including that node (brain area), SAL: salience network, SMN: somatomotor network, SUB: subcortical network, VAN: ventral attention network, VIS: visual network.

We next analyzed the masked edges from the AA model, as derived from a single‐fold (Figures 2 and S4). In the positive network, FPN between‐network connections were most featured, primarily between FPN and sensory networks as well as FPN‐DMN. There were some connections incorporating DMN, SAL, and SMN. High‐degree brain areas included the cerebellum, parietal areas, and dorsomedial prefrontal cortex. Circle plots from the FPN, in particular, show mostly cross‐hemispheric connections between prefrontal, motor areas, parietal areas, and limbic areas, with more diverse brain areas in the right hemisphere (Figure S5). The negative network (edges that negatively correlated with AA scores) contained connections within the SMN, and between the SMN and the VIS network, with some involvement of auditory and DMN networks. High degree nodes included somatomotor cortices, primary occipital cortices, and ventrolateral prefrontal cortex. Circle plots demonstrated dense connections across hemispheres between motor, parietal, temporal, visual, and insula areas (Figure S6).

### 3.4 Features in the NJ‐CPM

**FIGURE 3:**
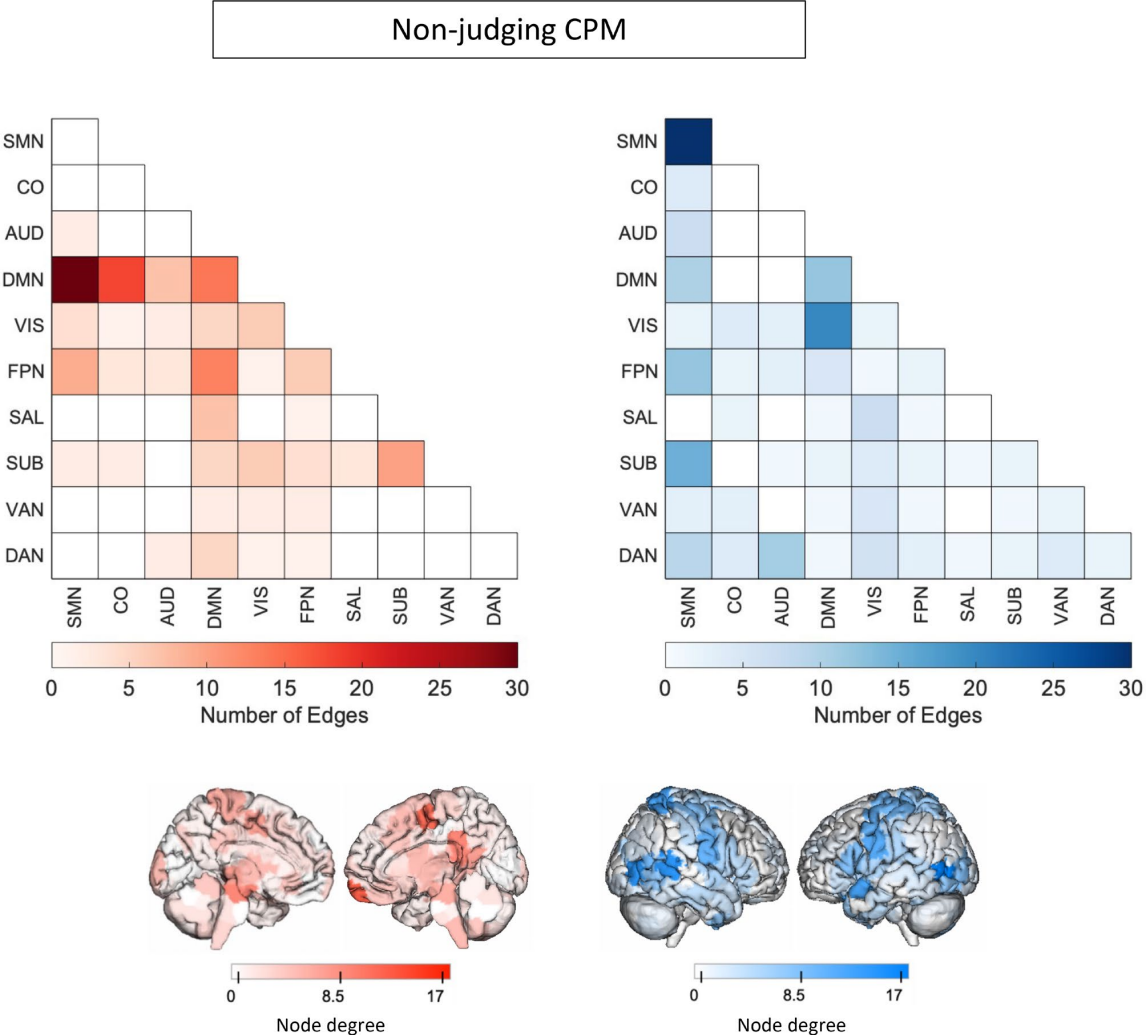
Edges included in single‐fold model of *Non‐judging* subscale of FFMQ using Shen atlas. In red are edges that positively predict *Non‐judging*. In blue are edges that negatively predict *Non‐judging*. Medial views are shown for positive network as high‐degree nodes are medial. AUD: auditory network, CO: cingular‐opercular network, DAN: dorsal attention network, DMN: default‐mode network, FPN: frontoparietal network, Node degree: number of connections including that node (brain area), SAL: salience network, SMN: somatomotor network, SUB: subcortical network, VAN: ventral attention network, VIS: visual network.

We analyzed the masked edges from the NJ model derived from a single fold (Figures 3 and S7). In the positive network, DMN connections to the rest of the brain were most featured, with some additional connections involving FPN and SUB. DMN‐SMN and DMN‐CO edges were most prevalent. High degree brain areas included the ventromedial prefrontal cortex, posterior cingulate cortex, and medial somatomotor areas. Circle plots of DMN connections show dense connections between left limbic areas and left motor areas, as well as the insula and parietal areas (Figure S8). The negative network (edges that negatively correlated with NJ scores) was widely distributed, with many edges in the SMN network, and between VIS and DMN. High degree brain areas included bilateral occipital areas, posterior temporal lobe including temporoparietal junction, and bilateral somatomotor areas. Circle plots demonstrated similar connections to the AA‐CPM negative network, with the exception of right subcortical involvement (Figure S9).

### 3.5 Testing Performance of AA‐CPM

**FIGURE 4:**
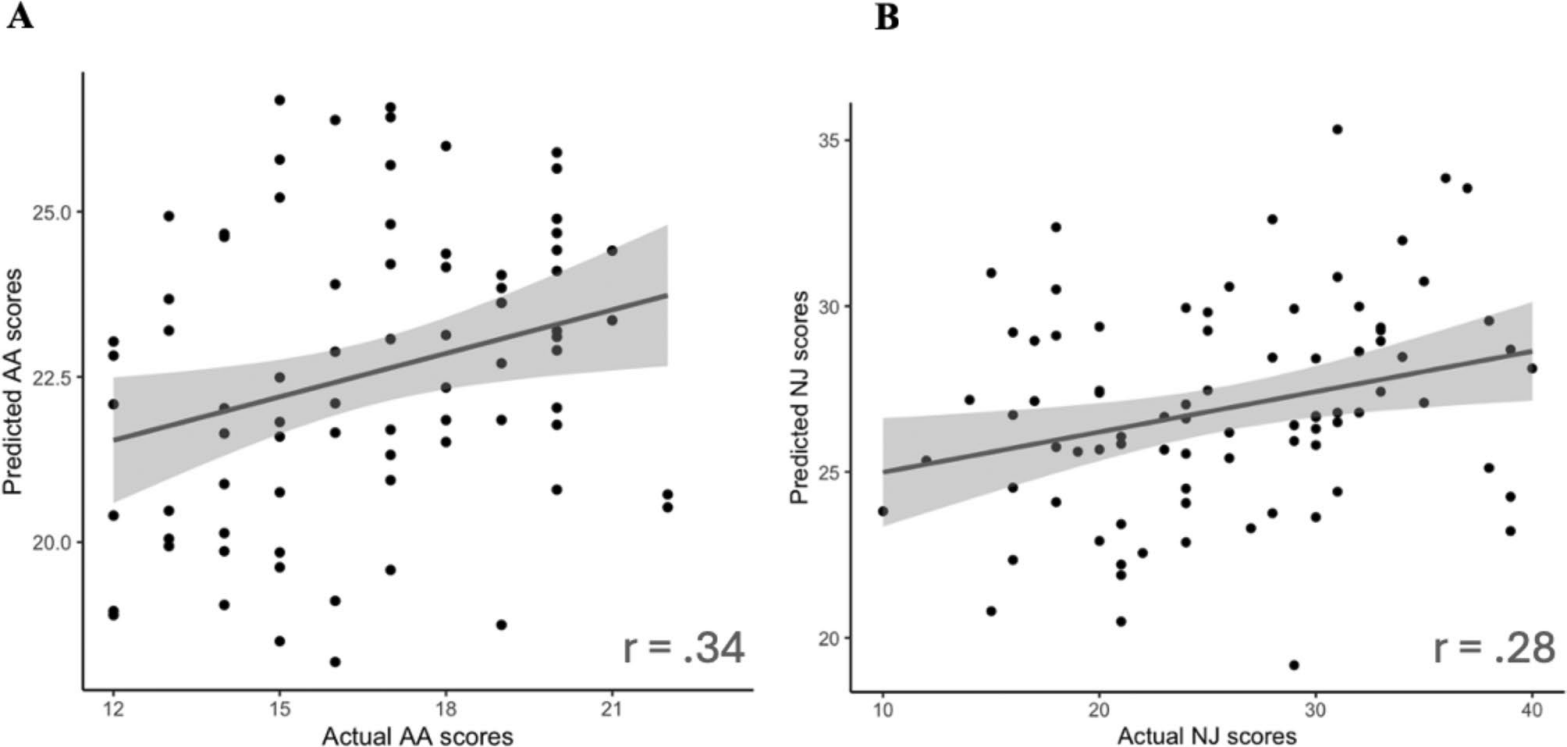
Test predictions vs. Actual scores. (A) Leipzig held‐out data prediction performance, for AA‐CPM predicting AA scores. Blue line is linear best fit, with gray 95% confidence intervals. (B) Stanford held‐out data prediction performance, for NJ‐CPM predicting NJ scores. Blue line is linear best fit, with gray 95% confidence intervals.

When we applied the AA‐CPM to the Leipzig dataset, we found a significant positive association between predicted and observed scores (*r*(75) = 0.34, *p* = 0.0025, two participants were removed due to head motion, MSE = 38.21) (Figure 4A). The association remained significant when partialling out head motion (*pr*(75) = 0.27, *p* = 0.020). The significant association was significant when examining only the negative network (*r*(75) = 0.33, *p* = 0.0031, MSE = 54.14), but not the positive network (*r*(75) = 0.032, *p* = 0.78, MSE = 232.96). We found no significant relationship between AA‐CPM predictions (positive, negative, and network strength) and observed AA scores in the Stanford dataset (*ps* > 0.5).

### 3.6 Testing Performance of NJ‐CPM

When we applied the NJ‐CPM to the Leipzig dataset, we found no association between predicted and observed scores (*ps* > 0.2, two participants were removed due to head motion). We found a positive relationship between NJ‐CPM predictions and observed NJ scores in the Stanford dataset (*r*(80) = 0.28, *p* = 0.012, MSE = 50.15) (Figure 4B), which was robust to partial correlations of clinical status (*r*(80) = 0.27, *p* = 0.013). The positive association was still present when just examining the negative network (*r*(80) = 0.27, *p* = 0.016, MSE = 49.66), but not the positive network (*r*(80) = 0.16, *p* = 0.16, MSE = 89.44). Results were identical when partialling out head motion.

### 3.7 Other Model Validation Steps

#### 3.7.1 Relationships Between AA‐CPM and NJ‐CPM

NJ and AA subscale scores were positively correlated in the Wisconsin training dataset (*r(*204) = 0.36, *p* < 0.001). In addition, there was a strong positive correlation between the AA‐CPM and the NJ‐CPM strengths across all datasets (*r*(345) = 0.66, *p* < 0.001). Twenty positive edges were shared between the models (2.02%, non‐parametric *p* < 0.001), and 58 negative edges were shared between the models (4.18%, non‐parametric *p* < 0.001). To evaluate the specificity of the AA‐CPM and NJ‐CPM, we examined whether they could cross‐predict in the held‐out datasets. The NJ‐CPM could predict AA in the Leipzig dataset (*r*(75) = 0.29, *p* = 0.0089). The AA‐CPM trended towards predicting NJ in the Stanford dataset (*r(*80) = 0.20, *p* = 0.065). Results were similar when just examining the negative networks. As a control, we examined whether a non‐significant model from the Wisconsin dataset (the Observing‐CPM), could predict in the hold out data. The Observing‐CPM did not predict AA in the Leipzig dataset (*r*(75) = 0.098, *p* = 0.39), nor NJ in the Stanford dataset (*r*(80) = 0.14, *p* = 0.22).

#### 3.7.2 Relationships With MW CPM

Trait mindfulness has been found to be negatively correlated with MW on tasks (Belardi et al. 2022; Mrazek, Smallwood, and Schooler 2012). We examined the correlation between overall network strengths for the AA‐CPM and NJ‐CPM and a previously published CPM for MW, the MW‐CPM (Kucyi et al. 2021), in data from all sites. There was a negative correlation between AA‐CPM and MW‐CPM, (*r*(345) = −0.22, *p* < 0.001) (Figure S10), as well as between NJ‐CPM and MW‐CPM (*r*(345) = −0.25, *p* < 0.001) (Figure S11). Overlaps between edges for AA‐CPM versus MW‐CPM and NJ‐CPM versus MW‐CPM were not significantly higher than chance (non‐parametric *p >* 0.1). The In the held out datasets, MW‐CPM negatively predicted NJ in the Stanford dataset (*r*(80) = −0.31, *p* = 0.0042), and no other predictions were statistically significant (*ps* > 0.5).

#### 3.7.3 Test–Retest Stability

We also examined the test–retest stability of the models in the Leipzig dataset, by comparing the network strengths for the first two runs to the last two runs using Pearson's correlations. The AA‐CPM and NJ‐CPM showed correlations (*r*(74) = 0.35, *r*(74) = 0.41) that were not more stable than random edges (non‐parametric *ps* > 0.5).

### 3.8 Sensitivity Analyses

In the combined, shuffled data, when accounting for score differences across sites using partial correlations, a model predicting NJ passed our selection threshold (non‐parametric *p* = 0.025), which generalized to the held‐out dataset (*r*(72) = 0.23, *p* = 0.05, MSE = 36.84). Generalization was reduced and non‐significant when removing the 22 Stanford individuals with clinical diagnoses (*r*(68) = 0.20, *p* = 0.11) although MSE was similar (35.33). No other models passed our selection threshold. It should be noted that the network models for the supplementary NJ model were similar to models found in the main NJ‐CPM (Figure S12). Results from the tangent parameterization of connectivity, Brain Basis Set regression, and Elastic Net Regression (Table S3) failed to generalize from Wisconsin to other datasets.

## 4 Discussion

We used connectome‐based predictive modeling (CPM) to investigate the relationships between trait mindfulness as measured by FFMQ, and functional networks across the whole brain (including the DMN, FPN, and SN) and assessed whether the relationships generalized to independent samples. Predictive modelling of functional connectivity networks has been used to predict meditation expertise (in 12 meditators; Guidotti et al. 2023), and distinguish participants before and after meditation training (in 25 undergraduates; Tang et al. 2017), but had previously not been applied to trait mindfulness. With 367 participants over three sites, this is the largest neuroimaging study of trait mindfulness to‐date (Treves, Pichappan, et al. 2024). While we did not find a generalizable model of total FFMQ scores, we did find models of the *AA* and *NJ* subscales that generalized to one of two independent datasets. The models showed highly similar negative networks (i.e., increasing connectivity negatively predicts mindfulness) involving DMN, VIS, and SMN connectivity, and these negative networks were responsible for generalization performance. Our findings highlight the importance of networks not investigated previously (e.g., VIS and SMN) (Treves, Pichappan, et al. 2024), inform new frameworks for defining trait mindfulness (Altgassen, Geiger, and Wilhelm 2023), and underscore the difficulty of using neuroimaging measures for predicting individual differences (Marek et al. 2022).

### 4.1 Networks Implicated in Trait Mindfulness

Previous studies on trait mindfulness have focused on seed‐based analysis of the triple networks: the DMN, FPN, and SN. A large cognitive neuroscience literature has implicated these networks in the regulation of external and internal attention (Buckner and DiNicola 2019; Menon 2011). In keeping with this, meta‐analytic reviews have found that mindfulness interventions lead to increases in DMN‐SN connectivity (Rahrig et al. 2022) and mindfulness practice (focused attention) leads to decreased activations in DMN regions like the posterior cingulate cortex compared to control conditions (Ganesan et al. 2022). Despite this, there is no consensus with regards to the networks' relationships to trait mindfulness. There are some indications of triple network involvement in predicting trait mindfulness in the current study. The predictive models developed here consist of positive networks (connectivity that predicts higher trait mindfulness) and negative networks (connectivity that predicts lower trait mindfulness). It is important to note that the models consist of hundreds of edges across the entire brain and here we summarize notable networks and regions. The positive network for the *AA* model included connections between FPN and other brain networks including sensory networks and DMN, with high‐degree nodes (regions involved in many connections) in the cerebellum, dorsomedial prefrontal cortex, and parietal cortex. FPN connectivity could be related to top‐down regulation of attention (Marek and Dosenbach 2018), and individuals who score high on *AA* may regulate their attention using the FPN. However, this connectivity did not generalize to predict scores in the test datasets. Instead, connections involving the DMN, SMN, and VIS networks negatively predicted mindfulness scores (both *AA* and *NJ*) in the training and test datasets (and under different training‐test splits of the data). Decreases in DMN connectivity with the rest of the brain could reflect differences in habitual self‐referential processing, for example, rumination (Butterfield, Grad‐Freilich, and Silk 2023; Frewen et al. 2020; Raichle et al. 2001; Zhou et al. 2020). The somatomotor (SMN) network has been implicated in MW (Kucyi et al. 2024; Mckeown et al. 2020; Vatansever et al. 2019). Speculatively, altered SMN connectivity may reflect different habitual processing of afferent thermo‐ceptive, proprioceptive or even pain signals, or it may reflect simulated motor action (Sormaz et al. 2018). Associations with visual network connectivity may reflect differences in sensory awareness, and visual network connectivity has been observed to change after mindfulness training (Kilpatrick et al. 2011).

Evidence that the models capture meaningful neural function is that they were significantly negatively correlated with a well‐established CPM (Kucyi et al. 2021). This is despite not involving the same brain connections. The MW CPM model was trained on self‐reported attention lapses during a task, and generalized to predict trait MW in additional samples. Individuals high in mindfulness may show reduced attentional lapses, for example, participants rating high on mindfulness questionnaires mind‐wander less (Mrazek, Smallwood, and Schooler 2012). Perhaps the mindfulness CPM models developed here could predict mindful states as well as traits. Future studies should explore MW and mindfulness and their relationships (Vago and Zeidan 2016) to brain measures in the same samples.

### 4.2 Distinctions Between Mindfulness Scales

The models that met our selection threshold in the training dataset were trained on *AA* and *NJ*. These two facets of mindfulness make up attitudinal and attentional components of mindfulness (Rau and Williams 2016) and are common in survey instruments measuring mindfulness (Altgassen, Geiger, and Wilhelm 2023). The *AA* subscale of the FFMQ is typically thought to reflect an attentional component of mindfulness (Baer et al. 2006). It involves questions from the MAAS (Brown and Ryan 2003), including “It seems I am running on automatic without much awareness of what I'm doing” (reverse‐coded). A network analysis found that *AA* clusters with the MAAS, MW questionnaires, and cognitive failures questionnaires (Beloborodova and Brown 2023). Finally, scores on *AA*, and the MAAS, respectively, correlate with objective behavioral measures of attention (Ching and Lim 2023; Mrazek, Smallwood, and Schooler 2012). Our study provides more external validation of self‐report mindful attention by identifying resting‐state connections across the whole brain that predicted *AA* scores.

The *NJ* subscale of the FFMQ is typically thought to reflect affective components of mindfulness, specifically one's tendency to become aware of thoughts and feelings without judgement (Baer et al. 2006). An example item is “I criticize myself for having irrational or inappropriate emotions” (reverse coded). Correlations between positive mental health outcomes and *NJ* are often found (Blanke, Riediger, and Brose 2018; Cortazar and Calvete 2019; Treves et al. 2023). One theorized link is through decreased rumination (Greco, Baer, and Smith 2011), or perseverating on negative self‐referential thoughts, memories, and one's own negative mood (Mennin and Fresco 2013; Nolen‐Hoeksema 1991).

One area of uncertainty highlighted by the current study is whether *AA* and *NJ* are distinct or whether they correspond to a single ontological concept of mindfulness. Standard definitions of mindfulness often unite attentional and attitudinal features of mindfulness, for example, mindfulness is a present‐focused attention, with an orientation of acceptance and non‐judgement (Bishop et al. 2004). Empirically however, this unity may not be supported. More recent research on self‐report mindfulness has indicated that single‐factor definitions of mindfulness may be neither accurate nor predictive of real‐world outcomes (Altgassen, Geiger, and Wilhelm 2023; Bednar, Voracek, and Tran 2020; Beloborodova and Brown 2023; Tran, Wasserbauer, and Voracek 2020). Our study contributes to this debate but does not resolve it. In the large training sample, there were discriminable brain connections (e.g., FPN vs. DMN) that positively predicted *AA* and *NJ*. However, the brain connections that predicted these subscales in the relatively smaller held‐out datasets were largely overlapping. It may be that large datasets are necessary to identify their distinctions neurally. It is also unclear why other subscales and the total FFMQ scores were not predictable. One possibility is that the shared variance between *AA* and *NJ* may be more predictable neurally than the FFMQ total scores which combine across 39 distinct items. Subscales like *Observing* are sometimes left out from measurement because they are understood differently by different populations (Baer, Gu, and Strauss 2022; Gu et al. 2016; Pang and Ruch 2019). An important consideration is that introspective ability confounds self‐reported mindfulness measurement (Grossman 2011), and more recent research has attempted to control for the reliability of responders before conducting correlations with functional neuroimaging measures (Kim et al. 2023b). To summarize, our results speak to the possibility of different operationalizations of mindfulness measurement—operationalizations for insight into self‐reported experiences may differ from operationalizations that align with objective measurement.

### 4.3 Limitations and the Difficulty of Individual Differences in Neuroimaging Research

We did not find complete model generalization. The negative network models (involving DMN, SMN, and VIS) predicted AA in the Leipzig dataset, and *NJ* in the Stanford dataset. One possibility is that the cross‐site differences were a barrier to generalization. The data acquisition parameters varied from site to site. Scanner parameters have an impact on activations and connectivity estimates (Friedman et al. 2008; Glover et al. 2012; Greve et al. 2013). In addition, the trait mindfulness scores varied significantly from site to site, and the connectivity features that predict mindfulness in one range of scores may not generalize to another range. It is unclear whether overall score differences between sites reflect real individual differences (Assumption 1) or noise (Assumption 2) (in which case they should be controlled for). In exploratory analyses, we tested the robustness of our models to this assumption, and even using partial correlations to control for score differences, the model predicting *NJ* still passed our selection threshold in the training set and generalized to the test split.

A second limitation concerns the reliability/stability of the CPM measures. A previous study found that when examining split‐halves of a 30‐min resting‐state scan, CPM networks were more reliable than individual edges (although edges showed a wide range of reliabilities with many exceeding that of CPM) (Taxali et al. 2021). We did not find this to be the case in our study. Reliability puts an upper limit on correlations between outcomes (Dubois and Adolphs 2016; Nunnally Jr 1970), and could have limited our ability to find meaningful relationships herein. In a recent study on dynamic functional connectivity, we found that only the most reliable brain measures showed significant relationships with trait mindfulness (Treves, Marusak, et al. 2024).

The predictive modeling results present some concerns as well. In addition to CPM, we conducted elastic net regression, Brain Basis Set regression, and a different connectivity parameterization (tangent‐space covariance). Although in the Wisconsin model‐training dataset the *AA* and *NJ* models showed significant correlations, these alternative predictive models did not generalize to the test datasets (nor did models for the other subscales). Our finding that only the CPM approach generalized to independent data highlights the sensitivity of the method while also suggesting some fragility of the brain–behavior relationships. An important caveat of the methods used in our study is that they do not involve any priors over the features used for prediction (a different approach is the network‐based statistics prediction toolbox [NBS‐Predict], which is biased towards finding connected sets of features; Serin et al. 2021).

A final limitation reflects the real‐world implications of these findings. Even though we combined connections across the whole brain for prediction, the amount of variance explained was low (~4%). This means that using the models for clinical prediction may not be feasible. This has been proposed to be a limitation of neuroimaging, not the models nor specific measures (Marek et al. 2022). Indeed, in the context of classifying individuals with depression, Winter et al. (2024) used multiple modalities of neuroimaging and tested millions of predictive models (with varying hyperparameters), showing a maximum accuracy of 62%. One source of this difficulty may be between‐individual variation in neural substrates. It may be the case that fMRI measures of functional connectivity are more powerful for predicting within‐individual variation, for example, fluctuations related to cognition, sleep, or arousal (Flournoy et al. 2024; Kucyi et al. 2024) or states of mindfulness versus inattentiveness (Weng et al. 2020).

### 4.4 Future Directions

Throughout our analyses, we explored the role of participant‐level factors in the model performance. There were small indications that diversity of participant populations affected our results. The Stanford testing dataset contained some individuals with anxiety and depression, and a supplementary sensitivity analysis showed that they were important for the *NJ* model's generalizability (but did not affect the main preregistered analyses). Some research has suggested that fMRI predictive models are not robust to participant diversity (Ellwood‐lowe, Whit, and Bunge 2021; Greene et al. 2022). In this case, as mindfulness is correlated negatively with depression and anxiety (Carpenter et al. 2019; Tomlinson et al. 2018), including these “diverse” participants may actually improve performance by widening the range of trait scores and brain function. Future studies could analyze common self‐report structure across anxiety, depression, and mindfulness scores before assessing brain relationships. Large‐sample studies should employ full measurement and reporting of the effects of participant cultural backgrounds, diagnoses, and development on brain–behavior relationships.

## 5 Conclusion

We conducted the largest neuroimaging study of trait mindfulness to‐date, with three independent fMRI datasets constituting 367 participants. We have demonstrated that subscales of trait mindfulness are to some degree represented within common whole‐brain patterns at rest. Future work could examine the discriminability of the brain representations and their malleability to mindfulness training.

## Ethics Statement

Procedures for the Wisconsin study were approved by the Healthy Sciences Institutional Review Board of the University of Wisconsin‐Madison. Procedures for the Leipzig study were approved by the ethics committee at the medical faculty of the University of Leipzig (097/15‐ff). The Stanford study was approved by the Stanford University Institutional Review Board.

## Conflicts of Interest

The authors declare no conflicts of interest.

## Supporting information


**Data S1.** Supporting Information.

## Data Availability

Preprocessed data and code will be uploaded to the following link upon publication: https://osf.io/tz86b/.
